# Supplementary material for: Infection with Helicobacter pylori Induces Epithelial to Mesenchymal Transition in Human Cholangiocytes
Source: Pathogens. 2020 Nov 21;9(11):971. doi: 10.3390/pathogens9110971 (PMC7700263; doi:10.3390/pathogens9110971)
Supplement: Supplementary file 1 [file pathogens-09-00971-s001.pdf]

Supplementary Materials

The following are available online at www.mdpi.com/xxx/s1,

Figure S1. TGF-β promoted nodule formation. TGF-β, an EMT-stimulant, induced nodule formation of H69 cholangiocytes. Cells were cultured in the presence of 5 ng/ml TGF-β in serum- and hormone-free medium for 24 hours. Micrographs at 0 (left) and 24 (right) hours; 5×magnification.

Figure S2. Size of colonies of cholangiocytes in soft agar**.** Colonies with a diameter ≥ 50 μm were counted and the diameter measured; mean values were 77.63, 91.62, 95.25 and 81.46 μm diameter at MOI of 0, 10, 50, and 100, respectively.

Figure S3. Growth of *Helicobacter bilis*-infected cholangiocytes as determined by the xCELLigence approach. H69 cells were exposed to increasing numbers of *H. bilis* (ATCC 43879) at MOI of 0, 10, 50, and 100. Cell growth was inhibited when compared to the uninfected cells, at all MOIs. Assay was performed in E-plates in minimal (serum-depleted) medium [97].

Table S1. Nucleotide sequences of primers specific for human EMT-associated and cancer stem cell marker genes.

Figure S1

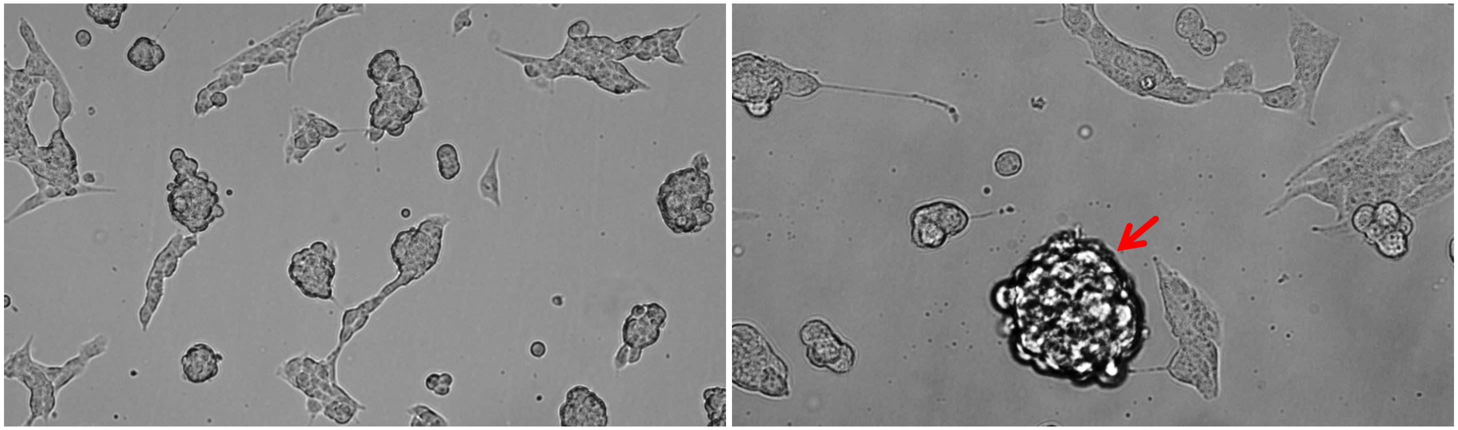

Figure S2

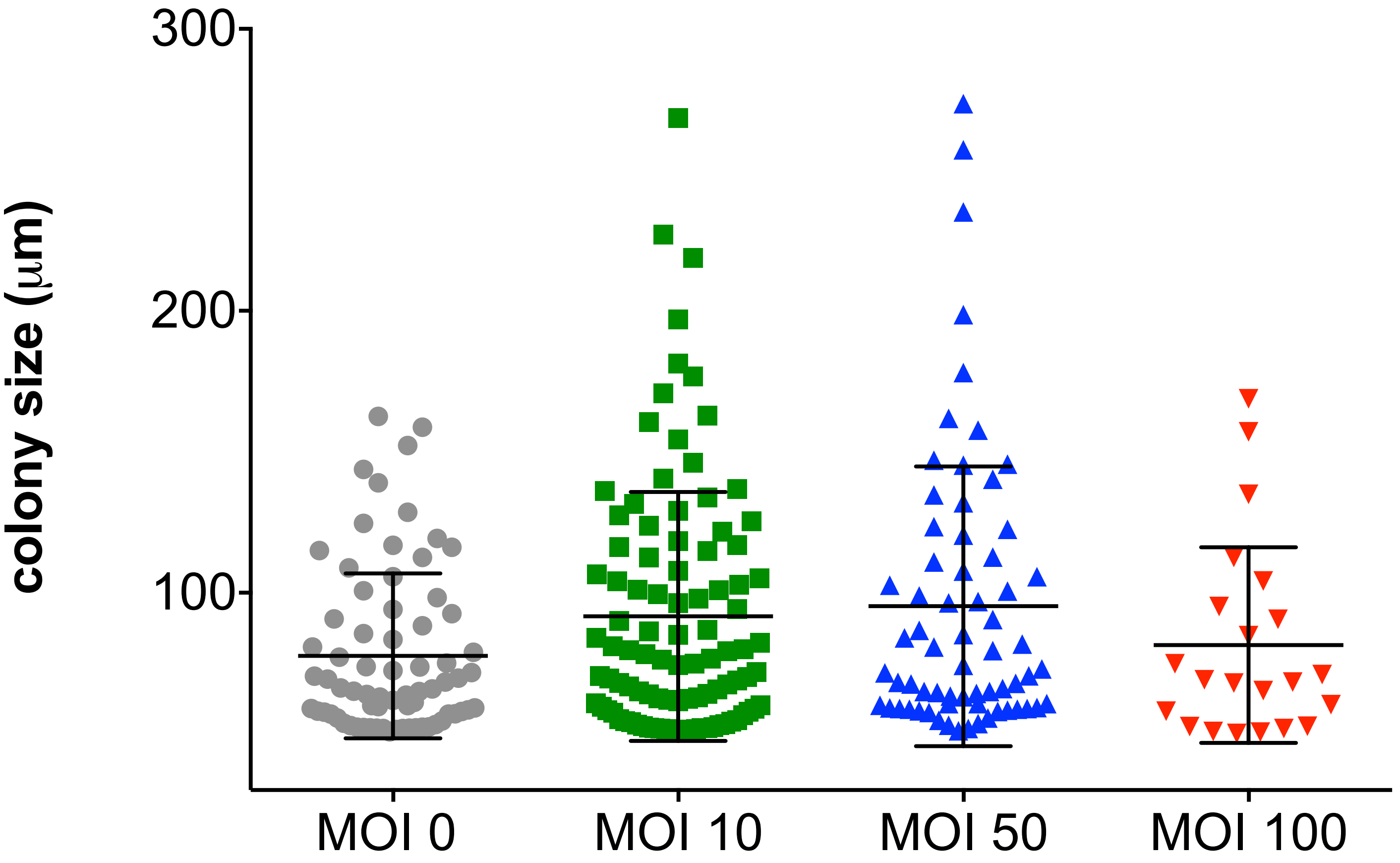

Figure S3

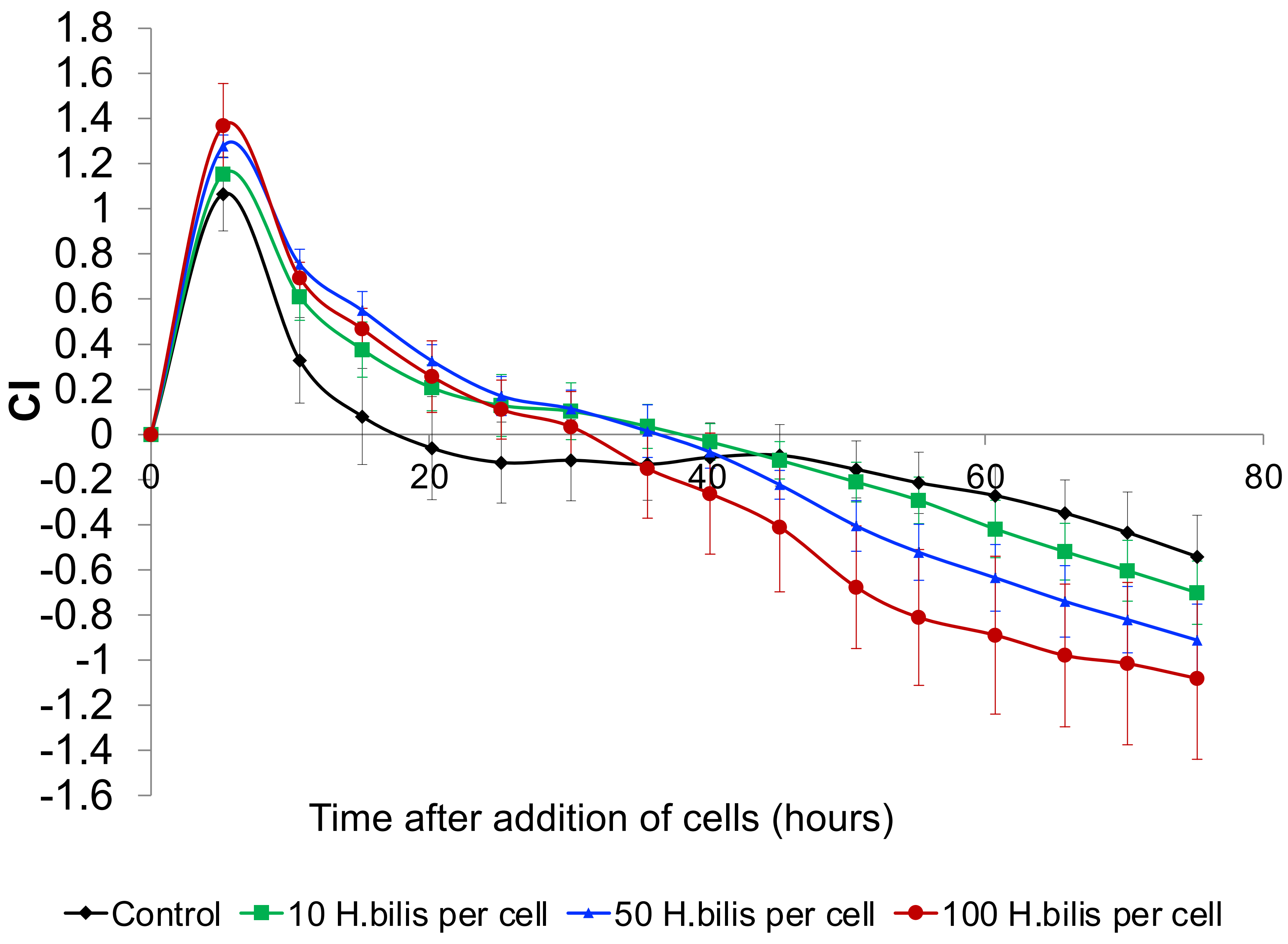
1.8
1.6
1.4
1.2
1
0.8
0.6
0.4
0.2
0
-0.2
-0.4
-0.6
-0.8
-1
-1.2
-1.4
-1.6
CI
0
20
40
60
80
Time after addition of cells (hours)
Control
10 H.bilis per cell
50 H.bilis per cell
100 H.bilis per cell

Table S1. Nucleotide sequences of primers specific for human EMT-associated and cancer stem cell maker genes.

Gene	GenBank accession	Primer sequence, 5'-3'	Amplicon size (bp)
CD24	NM_013230.3	Forward CACCCAGCATCCTGCTAGAC	259
		Reverse GAGACCACGAAGAGACTGGC	
CD44	NM_00610.3	Forward GGGAGTCAAGAAGGTGGAGC	237
		Reverse CTGAGACTTGCTGGCCTCTC	
F11r/Jam1	NM_016946.5	Forward GTGCCTACTCGGGCTTTTCT	201
		Reverse GAGCTTGACCTTGACCTCCC	
Gapdh	NM_002046.5	Forward CCCATGTTCGTCATGGGTGT	366
		Reverse TTCTAGACGGCAGGTCAGGT	
MMP7	NM_002423.5	Forward GGAGCTCATGGGGACTCCTA	172
		Reverse GGCCAAGTTCATGAGTTGCAG	
Snai1	NM_005985.3	Forward AGCTCTCTGAGGCCAAGGAT	325
		Reverse GACATTCGGGAGAAGGTCCG	
Vimentin	NM_003380.5	Forward GCAGGAGGCAGAAGAATGGT	216
		Reverse GCAGCTTCAACGGCAAAGTT	
Zeb1	NM_001128128.2	Forward CGCAGTCTGGGTGTAATCGT	477
		Reverse CCATGCCCTGAGGAGAACTG	
